# Corrigendum

**DOI:** 10.1080/17453674.2016.1189150

**Published:** 2016-05-16

Jaroma A, Soininvaara T, Kröger H. Periprosthetic tibial bone mineral density changes after total knee arthroplasty. ACTA ORTHOP 2016; 87 (3): 268-273.

http://dx.doi.org/10.3109/17453674.2016.1173982

When the above article was first published online, co-author Tarja Soininvaara’s surname was incorrectly spelt as Soinninvaara. This has now been corrected online and in the print edition.

